# Modified citrus pectin modulates splenic immune responses and galectin expression following cisplatin treatment in Wistar rats

**DOI:** 10.1007/s10735-026-10828-w

**Published:** 2026-05-13

**Authors:** Diego Dias dos Santos, Artur Francisco da Silva Neto, Laura Santana de Chiara, Mab Pereira Corrêa, Gisela Rodrigues da Silva-Sasso, José Marcos Sanches, Rinaldo Florencio-Silva, Lila Missae Oyama, Cristiane Damas Gil

**Affiliations:** 1 Department of Morphology and Genetics, Universidade Federal de São Paulo (UNIFESP), Rua Botucatu 740, Edifício Lemos Torres - 3° andar, São Paulo, 04023-900 SP Brazil; 2 Department of Physiology, Universidade Federal de São Paulo (UNIFESP), São Paulo, 04023-062 SP Brazil; 3 Center for Natural and Human Sciences (CCNH), Federal University of ABC (UFABC), Santo André, 09280-560 SP Brazil

**Keywords:** Cisplatin, Galectin, Immune remodeling, Immunotoxicity, Splenic toxicity, T cells

## Abstract

Modified citrus pectin (MCP), a polysaccharide from citrus fruits, modulates galectin-3 (Gal-3) and immune responses. Although MCP exhibits notable immunomodulatory effects, its role in drug-induced splenic and systemic toxicity remains unexplored. This study investigated the effects of MCP modulation on splenic immune remodeling and galectin expression in a rat model of cisplatin-induced toxicity. Wistar rats were divided into four groups (*n* = 5 animals/group): control (SHAM), MCP (100 mg/kg/day for 7 days), cisplatin (CIS, 10 mg/kg/day for 3 days), and MCP + CIS. Spleens were collected 6 h after the final cisplatin dose. Cisplatin induced splenic structural disorganization and selectively reduced nitric oxide levels without broadly affecting antioxidant enzymes. Cisplatin treatment was associated with increased CD3⁺ T cell labeling and enhanced tissue expression of Gal-1, -3, and − 9. MCP administration did not restore splenic architecture but promoted increased hemosiderin deposition in the red pulp, suggestive of enhanced erythrophagocytosis and altered iron handling, and markedly reduced CD3⁺ T cell immunoreactivity. MCP associated with cisplatin also reduced Gal-1 and Gal-3 levels and altered the relationship between galectins and splenic immune cell populations. Correlation analyses revealed positive associations between Gal-1, -3, and − 9 and CD68⁺ macrophages, as well as a selective association between Gal-3 and CD3⁺ T cells, exclusively in MCP + CIS animals. MCP does not mitigate cisplatin-induced splenic damage but alters immune cell distribution and galectin-related responses during cisplatin exposure.

**Supplementary Information:**

The online version contains supplementary material available at 10.1007/s10735-026-10828-w.

## Introduction

The spleen is an essential secondary lymphoid organ in the integration of the innate and adaptive immune systems. Its stroma is composed of a dense connective tissue capsule that projects septa, transporting vessels and nerves to the parenchyma. The reticular cells form an extracellular matrix of reticular fibers (type III collagen) that support the parenchyma. The white pulp contains follicular lymphoid tissue, while the red pulp houses splenic cords rich in macrophages, dendritic cells, and leukocytes (Mebius and Kraal 2005). The spleen plays a crucial role in red blood cell phagocytosis, iron recycling, and adaptive immune responses, and is vital for defending against hematological and immunological disorders (Lewis et al. 2019).

Cisplatin is a drug widely used in the treatment of various cancers. It acts by binding to DNA, forming cross-links that impair DNA synthesis, leading to cell cycle arrest and apoptosis. Its cytotoxicity is associated with oxidative stress and the generation of reactive oxygen species (ROS), mainly affecting mitochondria and compromising cell viability (Banerjee et al. 2018, Ghosh 2019). Studies in rats have shown that a single dose of cisplatin (5–6 mg/kg) causes structural disorganization in the spleen, with alterations in the white and red pulp areas, as well as changes in hemoglobin levels, leukocytes, and inflammatory factors such as tumor necrosis factor alpha (TNF-α) (Milićević et al. 1994, Abd El-Raouf et al. 2015). In mice, higher doses (10 and 20 mg/kg) of the drug caused additional damage, including hemosiderin deposition, ROS accumulation, and elevated levels of inflammatory cytokines (Banerjee et al. 2018).

Therapeutic strategies to protect vital organs such as the spleen are essential. Modified citrus pectin (MCP), a fruit-derived polysaccharide, has shown immunomodulatory, antiproliferative, and antimetastatic properties (Eliaz and Raz 2019, Caffall and Mohnen 2009). MCP, marketed as PectaSol-C, is easily absorbed in the intestine and has shown efficacy in inhibiting cancer progression, inflammation, and fibrosis (Eliaz et al. 2006). Studies indicate that MCP can modulate immune function by increasing the activity of macrophages, dendritic cells, and lymphocytes, as well as the production of pro-inflammatory cytokines such as interleukin (IL-17), interferon gamma (IFN-γ), and TNF-α (Merheb et al. 2019, Ramachandran et al. 2011, Duan et al. 2003).

MCP also antagonizes galectin-3 (Gal-3), the inhibition of which protects against cardiac inflammation, fibrosis, and renal apoptosis (Eliaz and Raz 2019). Preliminary studies suggest that MCP reduces Gal-3 and galectin-9 (Gal-9) levels in the liver in models of cisplatin toxicity, while increasing galectin-1 (Gal-1), possibly as a compensatory mechanism (Santos et al. 2023). However, the effects of MCP on the spleen still need to be investigated.

Galectins such as Gal-1, Gal-3, and Gal-9 are also crucial in regulating the differentiation and activation of lymphocytes and macrophages in the spleen. The absence of these proteins can lead to structural disorganization of the spleen and altered immune responses (Oliveira et al. 2018, Cao et al. 2018, Espeli et al. 2009, Smith et al. 2021). This study aims to investigate the effect of MCP on cisplatin-induced splenic toxicity, focusing on its immunomodulatory effects and regulation of galectins, and to determine whether MCP can be a therapeutic target for reducing splenic toxicity by inhibiting galectin-3 in pharmacological treatment with cisplatin.

## Materials and methods

### Animals

Male Wistar rats (250–350 g), aged 60–70 days, were housed under a 12 h/12 h light/dark cycle with a controlled temperature of 23–25 °C and *ad libitum* access to water and food. Animals were carefully acclimated for 7 days before the experiments to minimize stress. All procedures were approved by the Ethics Committee in Animal Experimentation of the Federal University of São Paulo (UNIFESP; CEUA number 5533211220) and performed in accordance with the protocols established by the National Council for the Control of Animal Experimentation (CONCEA).

## Experimental model of cisplatin-induced splenic toxicity

**Fig. 1:**
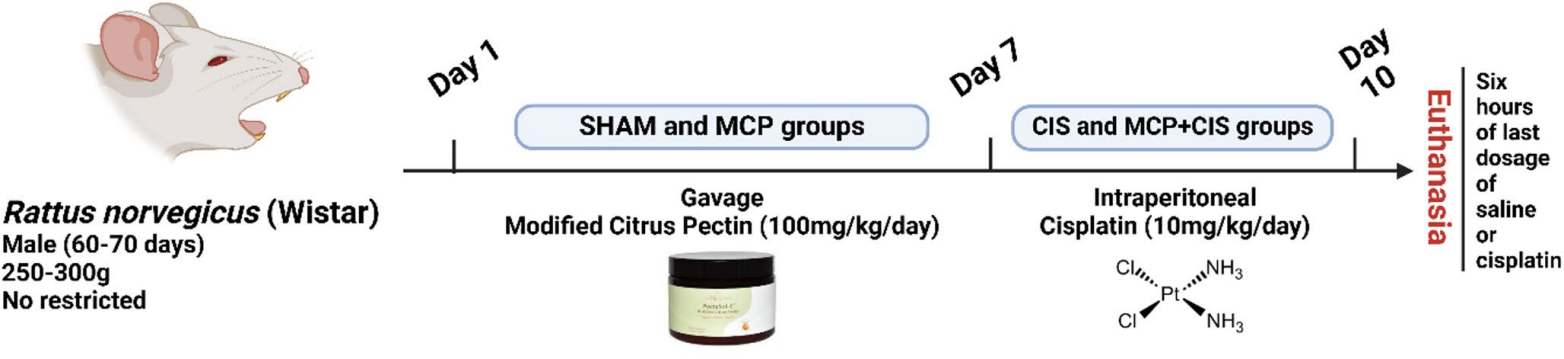
The experimental model of cisplatin-induced toxicity. The male rats were divided into four groups: SHAM, CIS, MCP, and MCP + CIS. On days 1–7, rats in the MCP and MCP + CIS groups received MCP (100 mg/kg/day) by oral gavage. On days 8–10, rats received i.p. injections of saline (SHAM and MCP groups) or cisplatin (10 mg/kg/day; MCP + CIS and CIS groups). Six hours after the final cisplatin injection, the rats were euthanized for spleen analysis

The animals were randomly distributed into four experimental groups (*n* = 5 animals/group): the SHAM (control) group received only sterile saline solution administration; the CIS group received intraperitoneal injections of cisplatin (10 mg/kg) on each of three days, totaling 30 mg/kg and the MCP group received 100 mg/kg/day of MCP (PectaSol-C, EcoNugenics, USA) by oral gavage for seven days, followed by intraperitoneal (i.p.) administration of saline; and the MCP + CIS group received 100 mg/kg/day of MCP for seven days, followed by i.p. administration of cisplatin. The doses of cisplatin and MCP used in this study were based on previous studies (Santos et al. 2023, Santos et al. 2024, Lucchi et al. 2022, Sena et al. 2022). Six hours after the last cisplatin administration, the animals were euthanized using isoflurane (BioChimico, Rio de Janeiro, Brazil), followed by decapitation immediately, and the spleens were harvested. Part of each spleen was frozen at -80 °C, while the remaining tissue was fixed and processed for histological and immunohistochemical analyses (Fig. 1).

## Histological and histochemical analysis

Spleen samples were fixed in 4% buffered paraformaldehyde for 48 h. The samples were then dehydrated in increasing grades of ethyl alcohol, diaphanized in xylene, and embedded in paraffin. Histological sections with a thickness of 4 μm were obtained using a rotary microtome (Leica RM2245), adhered to common slides for hematoxylin and eosin staining methods, for general morphology analysis; Perl’s method (Perl and Good 1992), for identifying and assessing the iron deposition; and Masson’s Trichrome, for identifying possible collagen deposition in the spleen.

To assess morphology, macrophage profile, and collagen deposition, the spleen sections were photographed using a 2.5x (Hematoxylin and Eosin), 10x (Masson’s Trichrome), and 20x (Perl’s method) objectives on a ZEISS AXIOSKOP 2 microscope at the Histology Laboratory, UNIFESP, São Paulo, SP. The areas of each white pulp region were calculated in mm^2^ using the Axiovision 4.9.1 image analyzer software (ZEISS). Densitometric analysis of the blue color in Perl and Masson’s Trichrome was performed using the freely accessible FIJI/ImageJ software (https://imagej.net/software/fiji/) with the Color Histogram plugin. The values are shown as mean ± S.E.M. of arbitrary units (a.u. - between 0 and 255).

## Immunohistochemical analysis

The expression of galectins (Gal-1, Gal-3, and Gal-9), CD68, and CD3 (macrophage and T cell markers, respectively) was examined in 4 μm histological sections of spleen tissue adhered to 4% silanized slides. Endogenous peroxidases were inactivated by incubation with 3% hydrogen peroxide for 20 min at room temperature in the dark. After washing with distilled water, the sections were incubated in citrate buffer, pH 6.0, at 96 °C for 60 min, then in 4% BSA in phosphate-buffered saline (PBS) at room temperature for 20 min. After washing in PBS, the sections were incubated overnight with rabbit polyclonal anti-Gal-1 (cat.#425800, Thermo Fisher Scientific, USA), mouse monoclonal anti-Gal-3 (cat.#MA1-940, Thermo Fisher Scientific, USA), anti-Gal-9 (cat.#ab153673, Abcam, USA), anti-CD68 (cat.#NB600-985, Novus Biologicals, Centennial, USA), diluted to 1:200 in 1% bovine serum albumin (BSA)/PBS, and rabbit polyclonal anti-CD3 ready-to-use (cat.# GA50361-2, Dako Omnis, USA). A negative control of the reaction was performed by omitting the primary antibodies. After washing, the sections were incubated with peroxidase-conjugated goat anti-rabbit or rabbit anti-mouse secondary antibodies (Thermo Fisher Scientific, USA) and detected using a colorimetric reaction with the substrate 3,3’-diaminobenzidine (DAB) (Dako, USA). Finally, the sections were counterstained with Carazzi’s hematoxylin. A negative control reaction was performed by omitting the primary antibody to confirm the specificity of immunoreactivity. Densitometric analysis of Gal-1, Gal-3, Gal-9, and CD3 was performed on spleen sections in a blinded manner using a 20x objective. Values are reported as the mean ± SEM of arbitrary units (a.u.). For CD68 analysis, the slides were scanned using the Aperio^®^ CS2 slide scanner (Leica Microsystems, Wetzlar, DE) using a 40x objective, and the positive cells were quantified using the QuPath software version 0.3.2 (University of Edinburgh, UK). Values are reported as the mean ± SEM of the percentage of positive cells of tissue per mm^2^.

## Activity of oxidant and antioxidant agents

Splenic homogenates were prepared using 0.1 g of sample in 1 mL of anhydrous potassium phosphate monobasic and dibasic buffer with ethylenediaminetetraacetic acid (EDTA) at pH 7.4, for use with the Beadblaster24 equipment (Benchmark Scientific, Inc.). The homogenates were incubated on ice and centrifuged at 16,000 ***×*** *g* for 10 min, and the supernatants were collected for biochemical analyses.

Lipid peroxidation was assessed by the TBARS assay, which detects thiobarbituric acid-reactive substances, with results expressed as malondialdehyde (MDA) equivalents as an indirect index of lipid peroxidation. Nitric oxide (NO) production was determined using the Griess reaction (Green et al. 1982). Catalase activity was evaluated by monitoring H₂O₂ decomposition, and superoxide dismutase (SOD) activity was determined based on its ability to inhibit pyrogallol oxidation (Marklund and Marklund 1974). Glutathione peroxidase (GPx) activity was measured using a commercial kit (Randox Laboratories Ltd., Crumlin, County Antrim, UK). The TBARS (MDA) and catalase assays were performed as previously described (Santos et al. 2023, Santos et al. 2024). Absorbance readings were obtained using microplate readers, and values were calculated after subtracting blanks and normalizing to tissue weight. Data are presented as mean ± SEM for MDA and NO (µM) and for enzymatic activities (U), expressed per mg of tissue.

### Statistical analysis

Data were analyzed using GraphPad Prism software version 9.3.0. The results were first subjected to descriptive analysis and normality testing (Kolmogorov–Smirnov test). For samples with normal distributions, a one-way analysis of variance (ANOVA) with Tukey’s post hoc test for multiple comparisons was applied. For samples with non-normal distributions, the Kruskal–Wallis test was performed, followed by Dunn’s post-hoc test. Pearson and Spearman correlation coefficients were calculated for pairwise comparisons of marker densitometry as determined by immunohistochemical analysis. In all cases, the p-values < 0.05 were considered statistically significant.

## Results

### Cisplatin induces disorganization of the splenic pulp and impairs the antioxidant defense system

**Fig. 2:**
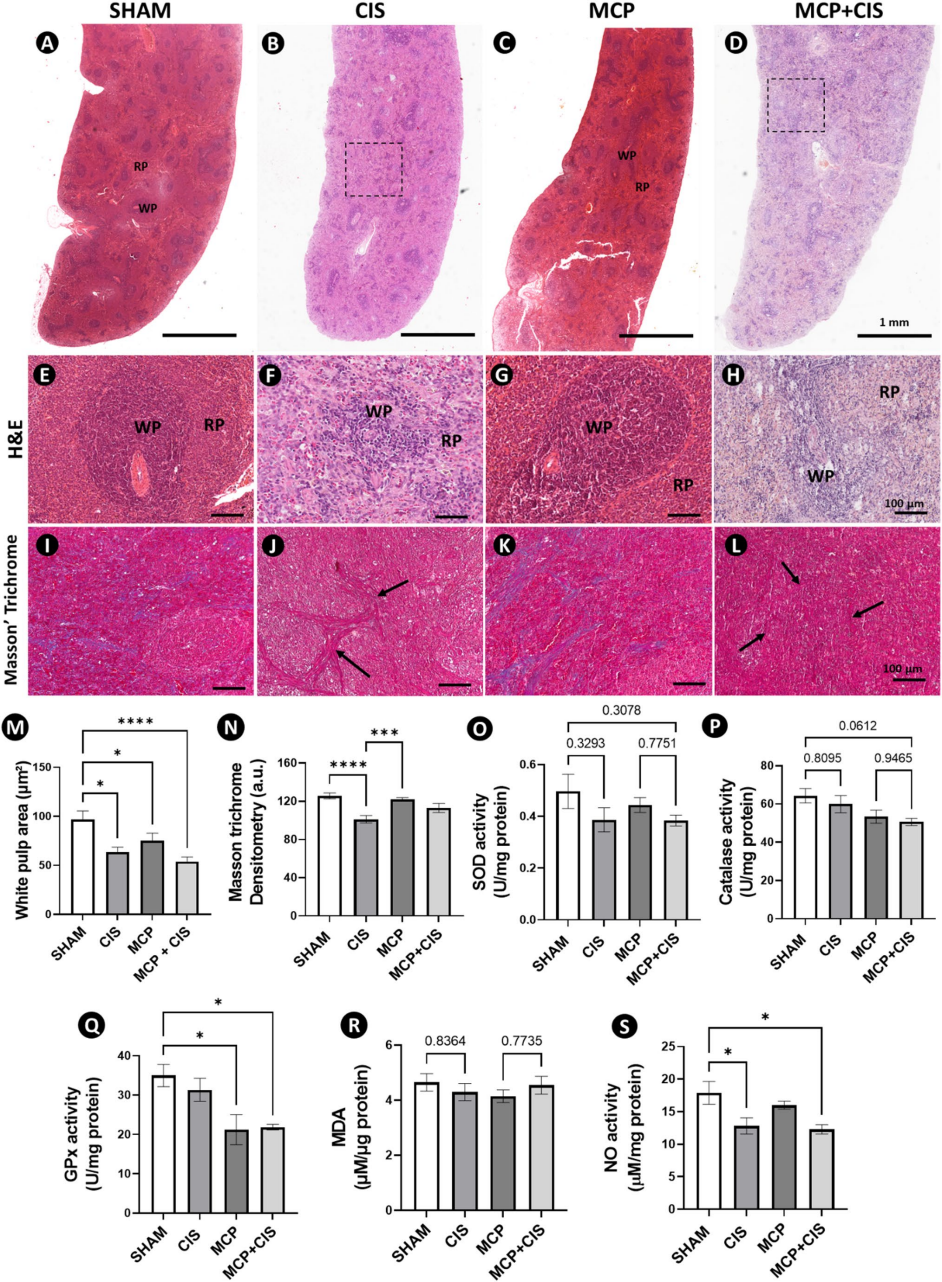
Deleterious effects of cisplatin on the spleen.**A**–**D**: Panoramic view of spleens. SHAM (A) and MCP (C) groups exhibit preserved organization of the white pulp (WP) and red pulp (RP), whereas CIS (B) and MCP + CIS (D) groups display evident disruption of splenic architecture with WP disorganization (dashed boxes). **E-H**: Higher magnification of spleens confirming preserved WP and RP organization in SHAM and MCP (E, G) and architectural disruption with disorganized splenic cords and WP in CIS and MCP + CIS (F, H). **I–L**: Collagen fiber distribution in the splenic parenchyma, showing greater collagen deposition in SHAM and MCP groups (E, G) and reduced deposition in CIS and MCP + CIS groups (F, H). Stains: H&E (A-D); Masson’s Trichrome (E-H). Scale bars: 1 mm (A-D); 100 μm (E-L). **M**: Proportion of white pulp area. **N**: Densitometric analysis of connective tissue content using Masson’s trichrome staining. **O-S**: Biochemical analyses of antioxidant and oxidative stress markers, including catalase, superoxide dismutase (SOD), glutathione peroxidase (GPx), malondialdehyde (MDA), and nitric oxide (NO). Data represent the mean ± SEM of white pulp area (%), Masson’s Trichrome densitometry (a.u.), catalase, SOD, GPx activity (U/mg protein), MDA, and NO (µM/mg protein). Dots in the bar graphs represent individual animals (*n* = 4 or 5 animals/group). *p < 0.05, ***p < 0.001, ****p < 0.0001 (I: Kruskal-Wallis followed by post-hoc Dunn test; J, M, O: ANOVA followed by post-hoc Tukey test)

In histological sections, the SHAM and MCP groups showed preserved splenic architecture, with well-defined white pulp (WP) and red pulp (RP) compartments (Fig. 2A, C, E, G). In contrast, cisplatin-treated groups (CIS and MCP + CIS) displayed evident histoarchitectural disruption, characterized by disorganization of splenic trabeculae and loss of the typical RP arrangement (Fig. 2B, D, F, H). These alterations are indicative of tissue remodeling consistent with cisplatin-induced cytotoxicity. Morphometric analysis revealed a significant reduction in WP area in the CIS group compared to SHAM (Fig. 2M), indicating marked impairment of splenic lymphoid compartments. Although no significant differences were detected between MCP and MCP + CIS groups, both showed a significant reduction in WP area relative to SHAM (Fig. 2M).

Masson’s trichrome staining revealed a significant reduction in collagen fiber deposition in the splenic parenchyma of the CIS group compared to both SHAM and MCP groups (Fig. 2I–L, N). In contrast, no significant differences were observed between the MCP and MCP + CIS groups (Fig. 2K, L, N).

Levels of oxidant and antioxidant markers were evaluated to assess redox status in the spleen. Regarding antioxidant agents, no significant differences were observed among groups for SOD and catalase activities. However, GPx activity was significantly reduced in both MCP and MCP + CIS groups compared to SHAM (Fig. 2Q). Regarding oxidant markers, no changes in MDA levels were observed (Fig. 2R). In contrast, NO levels were significantly reduced in the CIS and MCP + CIS groups compared to SHAM (Fig. 2S).

### MCP differentially modulates cisplatin-induced alterations in splenic phagocytic and immune cell profiles

**Fig. 3:**
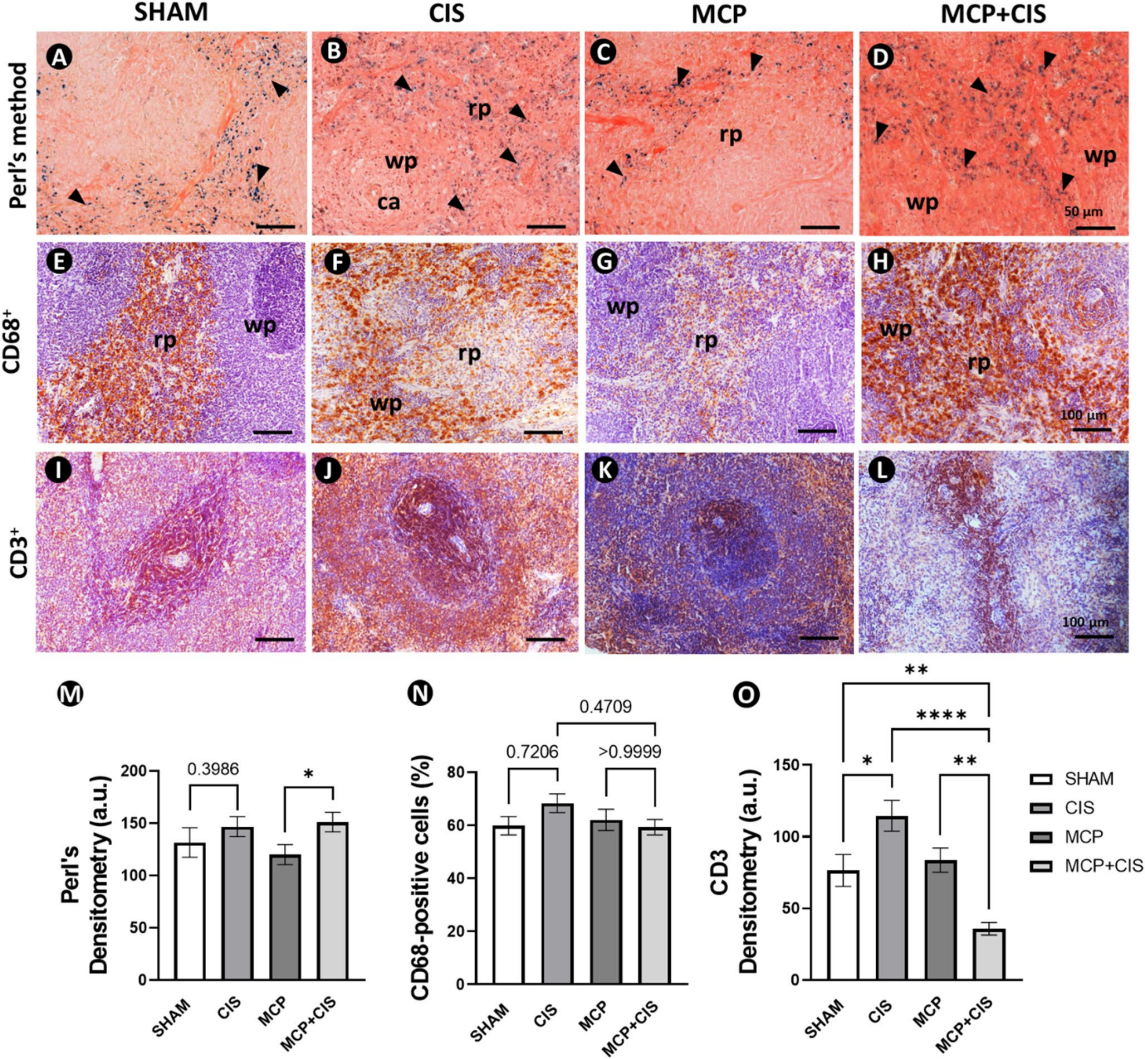
Splenic phagocytic and immune cell profile. **A**–**D**: Perls’ Prussian blue staining showing hemosiderin deposition (black arrowheads) in the spleen. Red pulp (rd). White pulp (wp). **E–H**: Immunohistochemical staining for CD68 showing macrophage in the spleen. **I–L**: Immunohistochemical analysis for CD3 showing the distribution of T cells between white and red pulp compartments. Experimental groups: SHAM (control animals), MCP (animals treated with MCP), CIS (animals treated with cisplatin), and MCP + CIS (animals treated with MCP and cisplatin). Counterstain: Carazzi’s hematoxylin. Scale bars: 50 μm (A-D); 100 μm (E-L). **M**: Densitometric analysis of Perls’ Prussian blue staining. **N**: Proportion of splenic CD68^+^ cells. **O**: Densitometric analysis of CD3 immunoreactivity. Data represent the mean ± SEM of Perls positivity (a.u.), CD68^+^ cells (%), and CD3 immunoreactivity (a.u.) (*n* = 5 animals/group). * *p* < 0.05, **p < 0.01; ****p < 0.0001 (M: t-test; N and O: ANOVA followed by post-hoc Tukey test)

The mechanisms of iron deposition and immune cell modulation, including CD68 and CD3, were evaluated to identify the alterations induced by cisplatin and the potential immunomodulatory effects of MCP. The SHAM and CIS groups showed no significant alterations in hemosiderin deposition, as assessed by Perls’ Prussian blue staining (Fig. 3A and B). In contrast, Perls’ staining revealed a significant increase in hemosiderin deposition in the MCP + CIS group when compared to the MCP group, predominantly within the red pulp (Fig. 3C and D), whereas no significant changes were observed in the CIS group relative to SHAM.

Immunohistochemical analysis for CD68, a macrophage marker, revealed a differential distribution of positive cells within the splenic compartments across experimental groups (Fig. 3E–H). In SHAM and MCP groups, CD68-positive cells were predominantly localized in the RP, with minimal staining observed in the WP. In contrast, CIS and MCP + CIS groups exhibited CD68-positive cells distributed in both RP and WP, indicating altered compartmental localization. Despite this distinct qualitative pattern, quantitative analysis of CD68 immunoreactivity did not show statistically significant differences among the groups (Fig. 3F, H, and N).

Immunohistochemical staining for CD3, a pan-T cell marker, revealed marked alterations in the distribution and expression of T cells within the spleen. CD3-positive cells displayed reduced localization within the white pulp, particularly around the central arteriole, concomitant with increased presence in the red pulp (Fig. 3I–L). Densitometric analysis demonstrated a significant increase in CD3 immunoreactivity in the CIS group compared to the SHAM group, whereas MCP + CIS animals exhibited a significant reduction in CD3 expression when compared with all other experimental groups (SHAM, CIS, and MCP) (Fig. 3O). Accordingly, the MCP + CIS group showed a pronounced decrease in CD3 labeling in both white and red pulp compartments (Fig. 3I–L).

## Differential modulation of galectin expression in the spleen following cisplatin and MCP treatment

To characterize the splenic expression pattern of galectins (Gal-1, Gal-3, and Gal-9) in response to cisplatin and MCP treatment, immunohistochemical and densitometric analyses were performed.

**Fig. 4:**
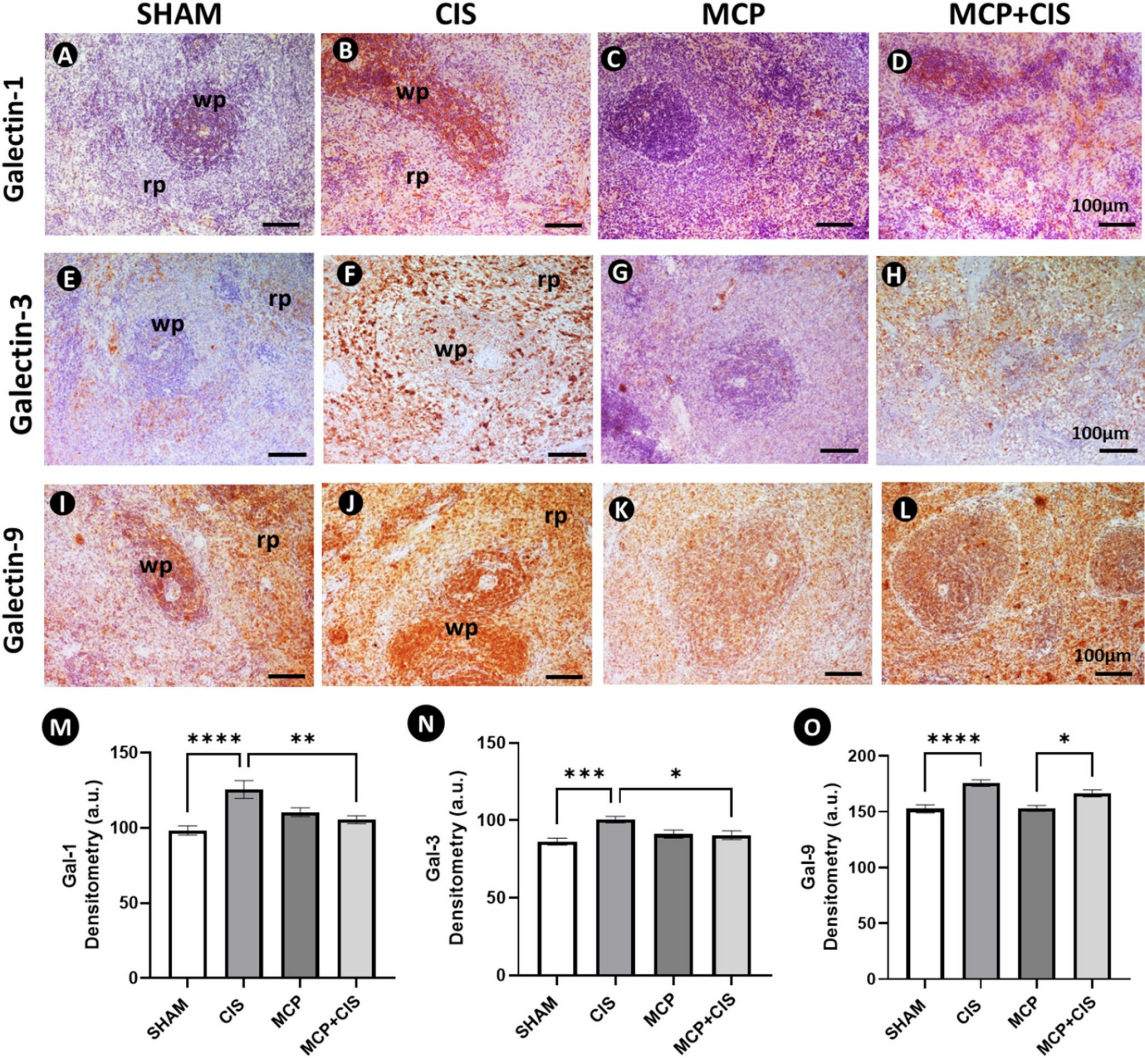
Analysis of galectin expression in the spleen.**A**–**D**: Galectin-1 (Gal-1) immunoreactivity. **E–H**: Galectin-3 (Gal-3) immunoreactivity. **I–L**: Galectin-9 (Gal-9) immunoreactivity. Immunolabeling is observed in both white pulp (wp) and red pulp (rp), with differences in distribution and intensity among groups: SHAM (control animals), MCP (animals treated with MCP), CIS (animals treated with cisplatin), and MCP + CIS (animals treated with MCP and cisplatin). Counterstain: Carazzi’s hematoxylin. Scale bars: 100 μm. **M–O**: Densitometric analysis of Gal-1, Gal-3, and Gal-9 immunoreactivity in splenic tissue. Data represent the mean ± SEM of arbitrary units (a.u.) of protein expression (*n* = 5 animals/group). * *p* < 0.05; ** *p* < 0.01, *** *p* < 0.001; **** *p* < 0.0001 (M, O, Q-S: ANOVA followed by post-hoc Tukey test; N: Kruskal-Wallis followed by post-hoc Dunn’s test)

Gal-1 and Gal-3 showed similar distribution patterns across experimental groups (Fig. 4A–H). In the SHAM and MCP groups, immunoreactivity was predominantly localized to the RP, whereas in the CIS and MCP + CIS groups, positive cells were also observed in the WP. In both cases, CIS was associated with increased immunoreactivity compared to SHAM and MCP + CIS, as confirmed by densitometric analysis (Fig. 4M, N). MCP treatment, alone or combined with CIS, reduced Gal-3 levels relative to CIS, returning to values comparable to SHAM (Fig. 4N).

Regarding Gal-9, immunohistochemical analysis showed a more dispersed distribution, with immunoreactive cells consistently observed in both WP and RP across all groups (Fig. 4I–L). Gal-9 immunoreactivity was more intense in the CIS and MCP + CIS groups than in the SHAM and MCP groups. These findings were corroborated by densitometric analysis, which demonstrated increased Gal-9 immunopositivity in CIS and MCP + CIS groups (Fig. 4O).

### Correlation between galectin expression and CD68⁺ macrophages and CD3⁺ T cells in the spleen

Subsequently, Pearson and Spearman correlation analyses were performed, based on data distribution, to evaluate the association between Gal-1, Gal-3, and Gal-9 and the populations of CD68^+^ macrophages and CD3^+^ T cells. These analyses aimed to determine whether changes in galectin expression were associated with alterations in the profile of these immune cell populations within the spleen.

**Fig. 5:**
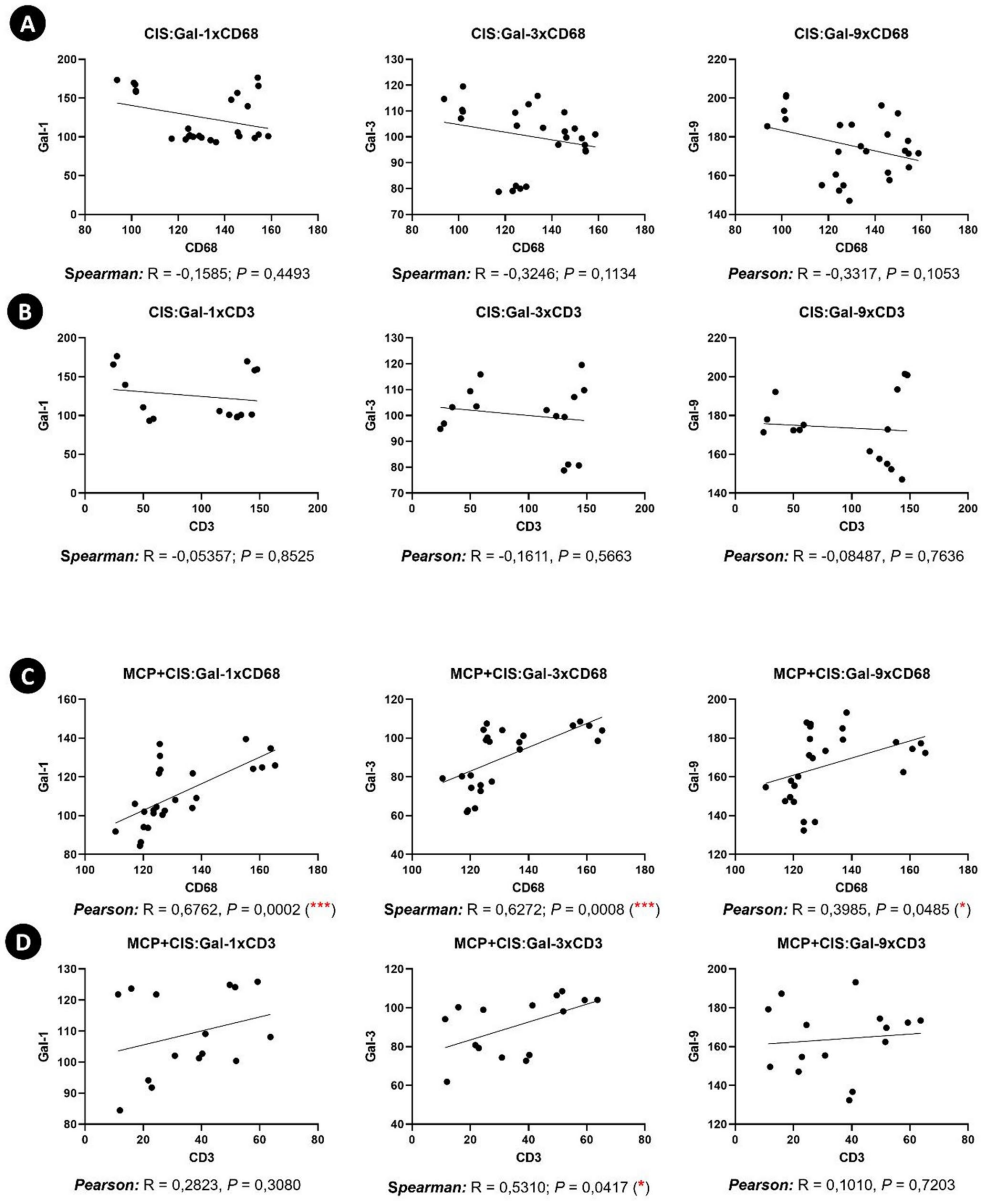
Correlation analysis of galectin expression and splenic immune cell populations between CIS and MCP + CIS groups.**A**,** B**: Correlation analyses show no significant correlations between galectin expression (Gal-1, Gal-3, and Gal-9) and CD68⁺ macrophages or CD3⁺ T cells in the CIS group. **C**: Correlation analysis indicating positive associations between Gal-1, Gal-3, and Gal-9 expression and the CD68^+^ macrophage population in the MCP + CIS group. **D**: Correlation analysis showing a positive association between Gal-3 expression and CD3⁺ T cells in the MCP + CIS group. Correlation analyses were performed using Pearson or Spearman tests, depending on the data distribution (*n* = 5 animals/group). *p < 0.05; ***p < 0.001; ****p < 0.0001

In animals from the SHAM and CIS groups, no significant correlations were observed between Gal-1, Gal-3, or Gal-9 expression and the numbers of CD68⁺ macrophages or CD3⁺ T cells (Fig. 5A, B; Supplementary Fig. S1A, B). In contrast, in the MCP and MCP + CIS group, positive correlations were detected between Gal-1, Gal-3, and Gal-9 expression and the CD68^+^ macrophage population (Fig. 5C; Supplementary Fig. S1C). Additionally, a positive correlation was observed exclusively between Gal-3 and CD3^+^ T cells in the MCP + CIS group (Fig. 5D), whereas no significant correlations were observed between galectin expression and the numbers of CD3⁺ T cells (Supplementary Fig. S1D).

## Discussion

Given that the role of Gal-3 in cisplatin-induced splenotoxicity remains unclear, this study sought to determine how pharmacologic Gal-3 inhibition with MCP affected the spleen in a Wistar rat model of cisplatin-induced toxicity. Our findings indicate that MCP does not exert a cytoprotective effect in this organ but rather promotes a context-dependent modulation of immune and molecular responses.

It is important to emphasize that cisplatin is an antineoplastic agent that, despite its widespread use in the treatment of various neoplasia (Ghosh 2019, Sadek et al. 2024, Coradini et al. 2007, Florea and Büsselberg 2011, Steenbrugge et al. 2022), can induce systemic toxicity affecting multiple vital organs, including liver (Santos et al. 2023, Abuzinadah and Ahmad 2020, Bakır et al. 2015, Bentli et al. 2013, Un et al. 2020, Qu et al. 2014), heart (Santos et al. 2024), kidneys (Jin et al. 2025, Kazak et al. 2024, Tekes et al. 2025, Abouzed et al. 2021), reproductive system (Sadek et al. 2024), central nervous system (El-Dein et al. 2026), and spleen (Banerjee et al. 2018, Milićević et al. 1994, Wang et al. 2010). These effects are highly tissue-specific and likely reflect differences in vascularization, immune composition, metabolic activity, and extracellular matrix (ECM) organization across organs. In this context, MCP is a promising candidate, as following esterification, pH change, and temperature-controlled enzymatic treatment, the small intestinal epithelium can absorb it and subsequently enter the bloodstream (Eliaz and Raz 2019). Consequently, oral administration of MCP appears to offer a homogeneous, systemic therapeutic strategy capable of addressing the multi-organ toxicity induced by cisplatin (Santos et al. 2023, Santos et al. 2024, Li et al. 2018, Xu et al. 2020, Wang et al. 2010).

Previous studies from our group have demonstrated that cisplatin induces systemic inflammation, including increased circulating neutrophils and elevated cytokine levels (IL-6 and IL-10) (Santos et al. 2023). Thus, the splenic alterations observed here should be interpreted within this broader systemic inflammatory framework. Consistent with this, our data demonstrate that cisplatin induces marked histoarchitectural disruption, with redistribution of immune cells and impairment of splenic compartmentalization, supporting a model of immune remodeling rather than simple immunosuppression (Banerjee et al. 2018, Wang et al. 2010, Khalaf et al. 2019, Lees et al. 2020).

The structural disorganization observed in the spleen may not be solely a consequence of cytotoxic injury but also reflect active remodeling driven by galectin-mediated immune regulation. Galectins, particularly Gal-1 and Gal-3, are known to regulate cell adhesion, migration, and immune cell compartmentalization within lymphoid tissues (Rabinovich et al. 1999, Fulcher et al. 2006, Ochieng et al. 1998). Thus, their increased expression following cisplatin exposure may contribute to the redistribution of immune cells between RP and WP, ultimately impacting splenic microarchitecture. In this context, the altered spatial organization of CD3⁺ T cells and CD68⁺ macrophages observed in this study may be functionally linked to galectin-dependent signaling pathways rather than being exclusively a passive consequence of tissue damage.

Regarding oxidative and nitrosative balance, cisplatin selectively reduced NO levels in splenic tissue without significantly altering SOD, catalase, GPx, or MDA levels. This finding suggests that cisplatin-induced splenic dysfunction is not associated with broad oxidative stress but rather with altered nitrosative signaling, which may impact vascular tone, immune cell trafficking, and splenic microcirculation (Tousoulis et al. 2012, Cyr et al. 2020). Experimental evidence indicates that cisplatin can modulate splenic NO production through nuclear factor-κB (NF-κB)–dependent inflammatory pathways and mitogen-activated protein kinase (MAPK) activation, contributing to endothelial dysfunction and immune dysregulation within the spleen (Banerjee et al. 2018).

In parallel with these biochemical changes, cisplatin treatment was associated with increased abundance of CD3⁺ T cells and enhanced tissue expression of Gal-1, Gal-3, and Gal-9, indicating an immune-activated splenic environment. From a functional perspective, this pattern is consistent with the known roles of galectins in regulating T cell activation, differentiation, and survival within secondary lymphoid organs (Rabinovich and Ilarregui 2009, Sundblad et al. 2017, Chou et al. 2009). The concomitant increase in galectin expression may represent a compensatory mechanism aimed at restraining excessive immune activation.

In contrast, MCP administration in cisplatin-treated animals did not restore splenic architecture. A potential explanation for this finding lies in the tissue-specific role of Gal-3 in extracellular matrix remodeling. In organs such as the liver and heart, Gal-3 is a well-established mediator of fibrosis, promoting fibroblast activation and collagen deposition (Chen et al. 2025, Calver et al. 2024). In these contexts, MCP-mediated Gal-3 inhibition is associated with antifibrotic and protective effects. However, the spleen exhibits a distinct stromal organization, primarily composed of reticular fibers and a highly dynamic immune cell network rather than a classical fibrotic ECM (Liakka et al. 1991). Therefore, Gal-3 inhibition in the spleen may not translate into structural recovery, as ECM remodeling is not the primary determinant of tissue organization in this organ. This distinction may explain why MCP fails to restore splenic architecture despite modulating galectin expression.

Instead, MCP treatment resulted in increased hemosiderin deposition in the red pulp, indicating enhanced erythrophagocytosis and altered iron metabolism. Notably, this effect was not observed in the CIS group alone, suggesting that MCP may potentiate iron accumulation under cisplatin-induced stress conditions. Cisplatin-induced disruption of iron homeostasis has been previously reported and is associated with erythrocyte injury and dysregulation of iron-related proteins (Wang et al. 2010). In this context, the increased hemosiderin content in MCP-treated animals may reflect exacerbated erythrocyte turnover or impaired iron recycling, rather than a protective response. These findings indicate that MCP shifts the splenic response toward iron dysregulation, which may contribute to tissue dysfunction instead of structural recovery.

Notably, MCP + CIS animals exhibited a pronounced reduction in CD3⁺ T cells, suggesting that MCP selectively modulates T cell maintenance or retention in the spleen under cisplatin exposure. These findings reinforce the concept that MCP acts primarily as an immunomodulatory agent rather than a cytoprotective compound. Conversely, studies using infliximab in methotrexate-induced splenic toxicity reported increased recruitment of CD3⁺ and CD68⁺ cells, highlighting that immune modulation in the spleen is highly context- and drug-dependent (Mercantepe et al. 2018).

A central finding of this study is the differential regulation of galectins by cisplatin and MCP. Cisplatin treatment markedly increased Gal-1 immunoreactivity in both white and red pulp compartments, whereas MCP associated with cisplatin significantly reduced Gal-1 protein levels. Gal-1 is known to regulate macrophage recruitment, promote M2 polarization (Perl and Good 1992), and modulate autophagy (Green et al. 1982). In T cells, Gal-1 functions as a negative immunoregulatory lectin, inhibiting T helper 1 (Th1) and Th17 differentiation, suppressing pro-inflammatory cytokine production, and inducing apoptosis of activated T cells, thereby contributing to immune tolerance and resolution of inflammation (Rabinovich and Ilarregui 2009, Sundblad et al. 2017, Hornung et al. 2017, Moreau et al. 2012). Within the context of cisplatin-induced splenic immune activation, increased Gal-1 expression may reflect a compensatory mechanism aimed at controlling excessive inflammation and lymphocyte activation. Thus, the reduction of Gal-1 in MCP + CIS animals may contribute to the observed decrease in splenic T cell abundance, without necessarily altering macrophage phenotype.

Similarly, Gal-3 expression exhibited a complex pattern. Although cisplatin increased Gal-3 tissue expression, MCP administration significantly reduced Gal-3 protein levels, consistent with its role as a Gal-3 inhibitor (Eliaz and Raz 2019, Xu et al. 2020, Hossein et al. 2019). Unlike Gal-1, Gal-3 generally exerts pro-inflammatory effects in T cell–mediated diseases, promoting T cell activation, dendritic cell-driven adaptive responses, and cytokine production (Radosavljevic et al. 2012, Breuilh et al. 2007). Gal-3 has been extensively studied as a mediator of tissue injury and fibrosis, particularly in cisplatin-induced nephrotoxicity, where MCP-mediated Gal-3 inhibition promoted tubular regeneration and reduced apoptosis (Li et al. 2018). However, previous work from our group demonstrated that MCP aggravated cisplatin-induced hepatic injury by increasing inflammatory cytokine levels, oxidative stress, and mitochondrial dysfunction, while also altering the balance of other galectins (Santos et al. 2023).

In this context, Gal-3 appears to exert tissue-specific functions. In the liver, it plays a cytoprotective role, particularly through the regulation of mitochondrial integrity and oxidative stress, whereas in the spleen its modulation was not associated with preservation of tissue architecture, suggesting a distinct functional role. These findings indicate that the effects of Gal-3 inhibition depend on the cellular and microenvironmental context, likely reflecting differences between parenchymal organs and secondary lymphoid tissues. Taken together, Gal-3 inhibition may attenuate inflammatory signaling in some settings while exacerbating tissue damage in others, depending on local immune and metabolic conditions.

Gal-9 displayed a distinct regulatory profile. While cisplatin increased Gal-9 expression, MCP + CIS animals exhibited a further increase in Gal-9 protein levels, suggesting compensatory or counter-regulatory mechanisms. Gal-9 plays a critical role in T cell regulation through interactions with programmed cell death protein 1 (PD-1) and T-cell immunoglobulin and mucin-domain containing-3 (TIM-3) (Castillo-González et al. 2021)and has been associated with immune tolerance, fibrosis, and autoimmunity (Zhang et al. 2021). Increased Gal-9 expression may therefore contribute to the reduction in splenic T cell abundance observed in MCP + CIS animals.

Correlation analyses revealed associations between galectins and immune cells only in MCP + CIS animals. While cisplatin-treated animals did not show significant associations between galectin expression and immune cell populations, MCP + CIS animals exhibited strong positive correlations between Gal-1, Gal-3, and Gal-9 and CD68⁺ macrophages, as well as a selective association between Gal-3 and CD3⁺ T cells. These findings suggest that MCP not only alters galectin levels but also reshapes their relationship with splenic immune cell populations, reinforcing its role as an immunomodulatory agent rather than a cytoprotective compound.

This study presents some limitations that should be considered when interpreting the findings. The sample size (*n* = 5 animals/group), although consistent with previous experimental studies, may limit the statistical power to detect subtle differences, particularly in parameters that did not show significant changes. In addition, correlation analyses were performed in relatively small groups and should therefore be interpreted with caution. Another aspect to consider is that only male animals were included, which may limit the extrapolation of these findings to females, given potential sex-related differences in immune responses. Finally, the experimental design focused on an acute exposure model, which may not fully reflect long-term effects of cisplatin or MCP treatment.

Taken together, these findings indicate that galectin signaling plays an active role in shaping splenic immune organization under cisplatin exposure and that MCP modulates this process in a tissue-specific and context-dependent manner.

## Conclusion

Cisplatin induces splenic immune remodeling marked by architectural disruption, T cell redistribution, and increased galectin expression. MCP does not restore these alterations but modulates immune responses, particularly T cell dynamics, iron handling, and galectin signaling, acting as a context-dependent immunomodulator rather than a cytoprotective agent. These findings highlight the tissue-specific nature of galectin-mediated effects and support the need for organ-oriented interpretation of galectin-targeted therapies.

## Supplementary Information

Below is the link to the electronic supplementary material.

Supplementary Material 1

## Data Availability

Data will be made available on request.

## Declarations

### Generative AI use

During the preparation of this work, the author used Grammarly Plagiarism AI, ChatGPT (version 5.1), and Microsoft Word Editor to translate, correct grammatical errors, organize information, and identify plagiarism. After using these tools/services, the authors reviewed/edited all content as needed and assume full responsibility for the published article’s content.
