# Supplementary material for: Modified citrus pectin modulates splenic immune responses and galectin expression following cisplatin treatment in Wistar rats
Source: J Mol Histol. 2026 May 13;57(3):165. doi: 10.1007/s10735-026-10828-w (PMC13171650; doi:10.1007/s10735-026-10828-w)
Supplement: Supplementary file 1 — Supplementary Material 1 [file 10735_2026_10828_MOESM1_ESM.docx]

**Modified citrus pectin modulates splenic immune responses and galectin expression following cisplatin treatment in Wistar rats**

Diego Dias dos Santos^1^; Artur Francisco da Silva Neto^2^; Laura Santana de Chiara^1^; Mab Pereira Corrêa^1^; Gisela Rodrigues da Silva-Sasso^1^; José Marcos Sanches^3^; Rinaldo Florencio-Silva^1^; Lila Missae Oyama^2^; Cristiane Damas Gil^1*^.

**Table of Contents**

- **Supplemental Figure 1.** **Correlation analysis of galectin expression and splenic immune cell populations between SHAM and MCP groups**.


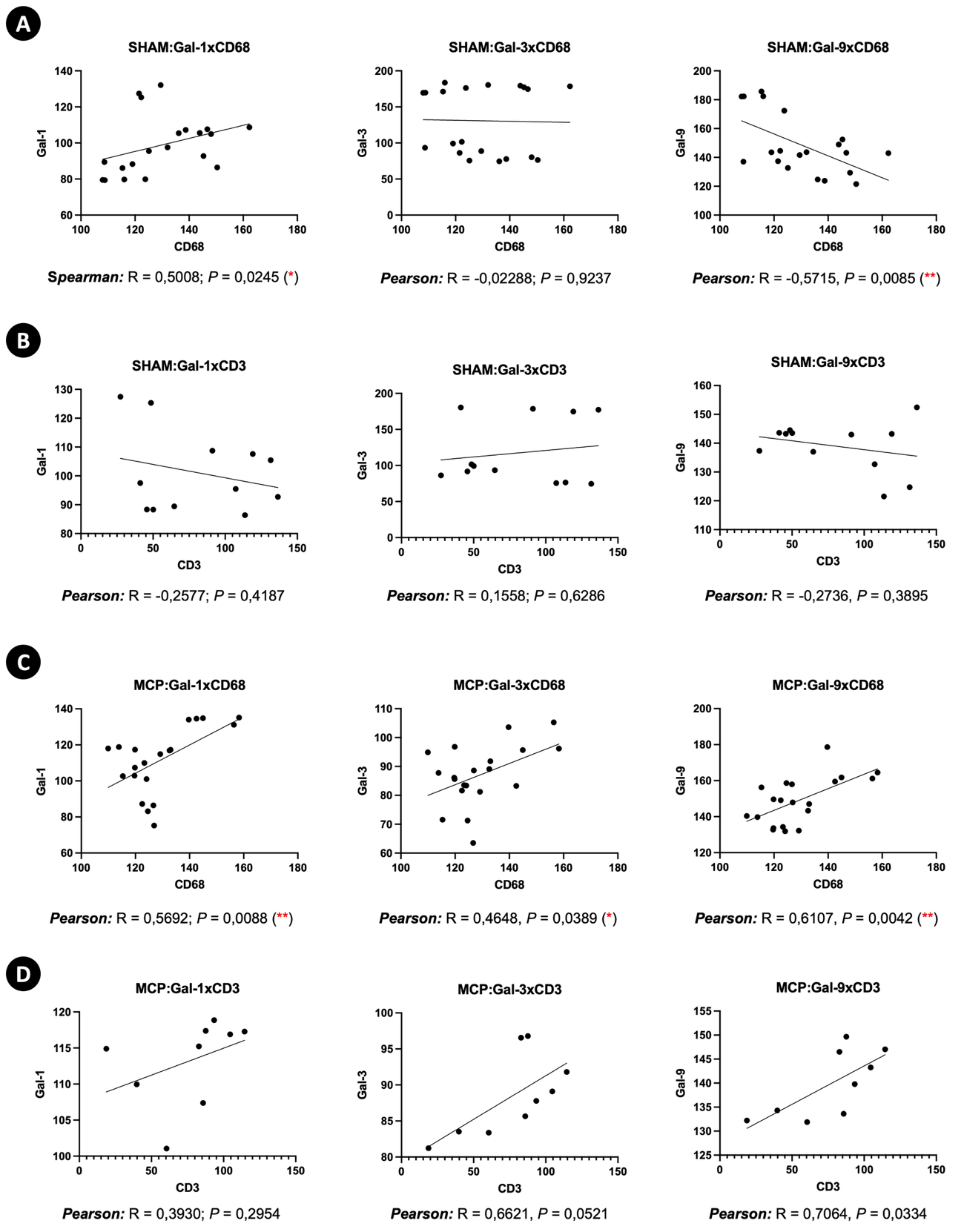


**Supplemental Figure 1. Correlation analysis of galectin expression and splenic immune cell populations between SHAM and MCP groups. A:** Correlation analysis showing a positive association between Gal-1 expression and CD68+, and a negative association between Gal-9 expression and CD68+ in the SHAM group. **B**: Correlation analyses show no significant correlations between galectin expression (Gal-1, Gal-3, and Gal-9) and CD3⁺ T cells in the SHAM group. **C:** Correlation analysis indicating positive associations between Gal-1, Gal-3, and Gal-9 expression and the CD68^+^ macrophage population in the MCP group. **D:** Correlation analyses show no significant correlations between galectin expression (Gal-1, Gal-3, and Gal-9) and CD3⁺ T cells in the MCP group. Correlation analyses were performed using Pearson or Spearman tests, depending on the data distribution (n = 5 animals/group). **p* < 0.05; ***p* < 0.01.
